# Supplementary figures and images for: Antigenic diversity in malaria parasites is maintained on extrachromosomal DNA
Source: bioRxiv. 2023 Feb 2:2023.02.02.526885. Preprint. [Version 1] doi: 10.1101/2023.02.02.526885 (PMC9915586; doi:10.1101/2023.02.02.526885)

**a**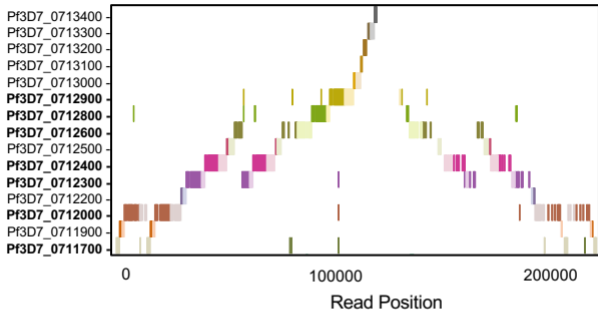**b**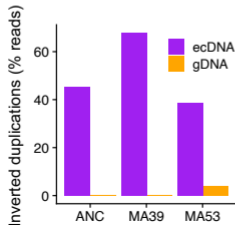**c**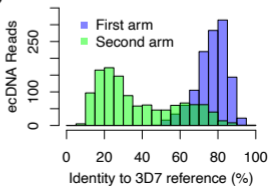

Supplement: Supplement 1 — Extended Data Fig. 1: Structural variation among assembled PacBio contigs. a, Genome-wide dot plots. Insets are internal var with >1 contig. b, Summary of loci with >1 contig across assemblies. c, Visualization of indel polymorphism across contigs at two internal var loci on chr4. Extended Data Fig. 2: Molecular confirmation of long-read polymorphism. a, PCR of breakpoints observed in contigs that map to the second internal var locus on chr12. Colored bands represent amplicons. Asterisks indicate the amplicons expected in each sample, based on assembled PacBio contigs. In the left diagram, the pink amplicon is expected to be 562 bp in the reference allele (e.g. allele A) and 667 bp with the gene conversion from PF3D7_0700100 (e.g. allele B). These data confirm the existence of breakpoints detected with PacBio in ANC and MA53 but undetected in MA39 and MA47. b, PCR detection of allele F. Asterisks indicate the amplicons expected in each sample, based on Nanopore reads. c, Southern blot of copy number variation in PF3D7_1240300. Teal asterisks mark the bands expected in each sample, based on Nanopore reads. Extended Data Fig. 3: Structural polymorphism across Nanopore reads mapping to internal var loci from all clonal MAL. Extended Data Fig. 4: Genome-wide coverage of extrachromosomal, circular DNA relative to genomic DNA. Extended Data Fig. 5: Large inverted duplications on ecDNA reads. a, Example read from MA54 containing a large inverted duplication. b, Plasmid-Safe-treated DNA is strongly enriched for “triangle reads”. c, Signal degradation consistent with single-strand annealing after passing through Nanopore. Supplementary Table 1. Summary of PacBio assemblies including PAF alignments to 3D7. Supplementary Table 2. ddPCR count data from QuantaSoft. Supplementary Table 3. Structural variation on Nanopore reads assigned to non-hypervariable var loci. The three events fixed in ANC and all MAL are considered wild-type. [file media-1.zip › supplement_biorxiv_020223/EDF5-Triangles.pdf]

**a**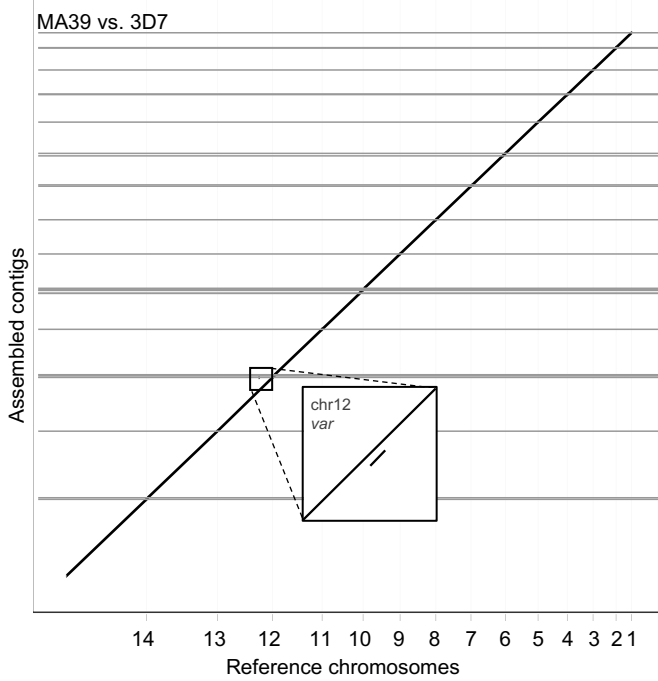**MA47 vs. 3D7**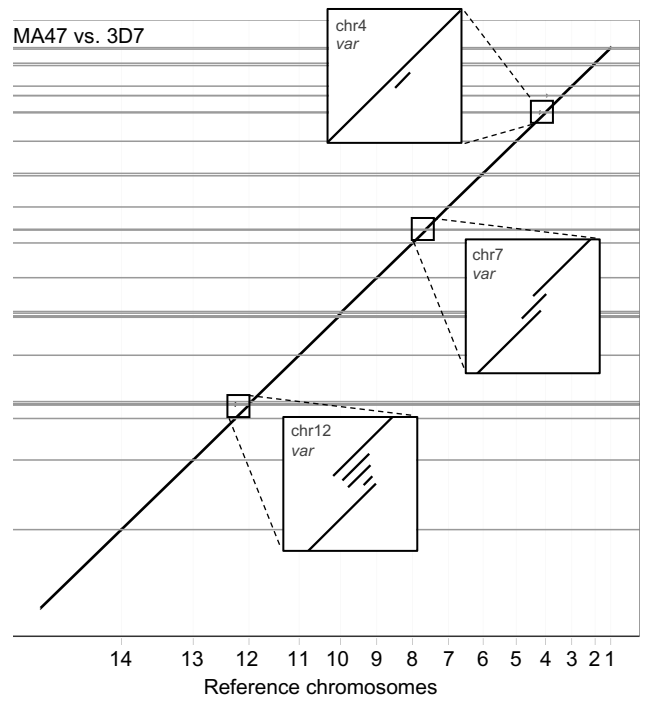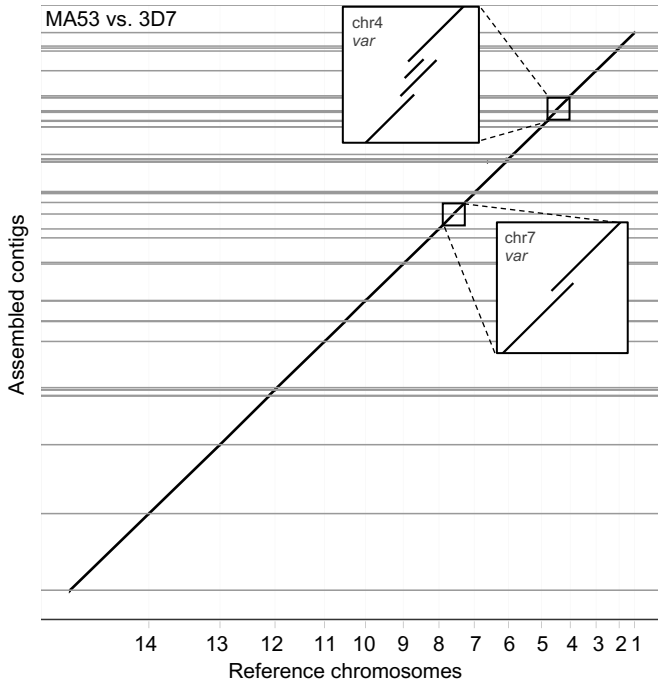**b**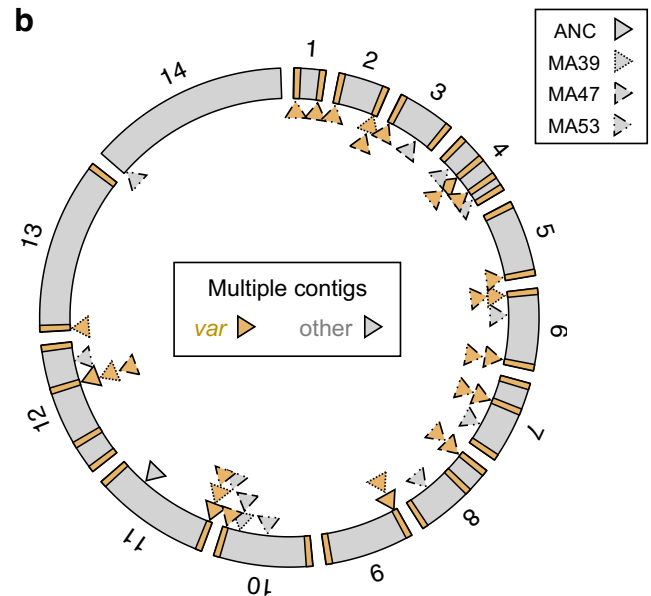**c****3D7 Reference – chr4int1**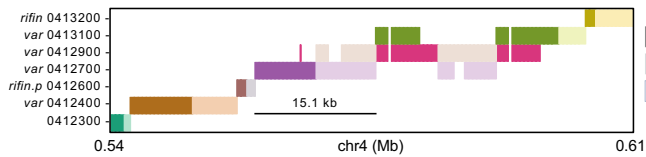**e****3D7 Reference – chr4int2**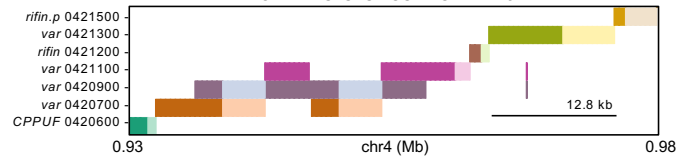**d** **ANC contigs**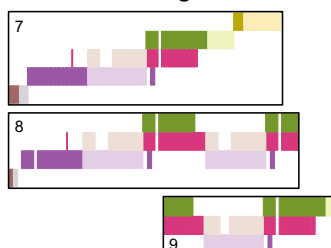**f** **MA47 contigs**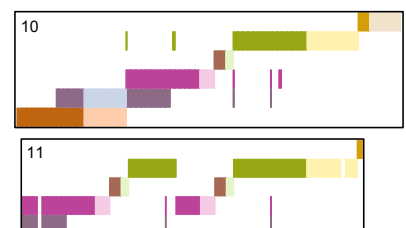

Supplement: Supplement 1 — Extended Data Fig. 1: Structural variation among assembled PacBio contigs. a, Genome-wide dot plots. Insets are internal var with >1 contig. b, Summary of loci with >1 contig across assemblies. c, Visualization of indel polymorphism across contigs at two internal var loci on chr4. Extended Data Fig. 2: Molecular confirmation of long-read polymorphism. a, PCR of breakpoints observed in contigs that map to the second internal var locus on chr12. Colored bands represent amplicons. Asterisks indicate the amplicons expected in each sample, based on assembled PacBio contigs. In the left diagram, the pink amplicon is expected to be 562 bp in the reference allele (e.g. allele A) and 667 bp with the gene conversion from PF3D7_0700100 (e.g. allele B). These data confirm the existence of breakpoints detected with PacBio in ANC and MA53 but undetected in MA39 and MA47. b, PCR detection of allele F. Asterisks indicate the amplicons expected in each sample, based on Nanopore reads. c, Southern blot of copy number variation in PF3D7_1240300. Teal asterisks mark the bands expected in each sample, based on Nanopore reads. Extended Data Fig. 3: Structural polymorphism across Nanopore reads mapping to internal var loci from all clonal MAL. Extended Data Fig. 4: Genome-wide coverage of extrachromosomal, circular DNA relative to genomic DNA. Extended Data Fig. 5: Large inverted duplications on ecDNA reads. a, Example read from MA54 containing a large inverted duplication. b, Plasmid-Safe-treated DNA is strongly enriched for “triangle reads”. c, Signal degradation consistent with single-strand annealing after passing through Nanopore. Supplementary Table 1. Summary of PacBio assemblies including PAF alignments to 3D7. Supplementary Table 2. ddPCR count data from QuantaSoft. Supplementary Table 3. Structural variation on Nanopore reads assigned to non-hypervariable var loci. The three events fixed in ANC and all MAL are considered wild-type. [file media-1.zip › supplement_biorxiv_020223/EDF1-PacBio.pdf]

### 3D7 Reference

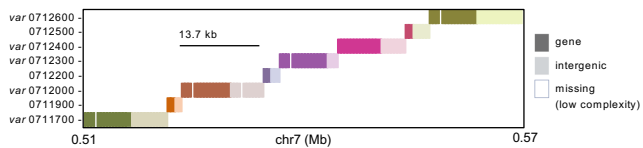

### 'Clonal' MA lines

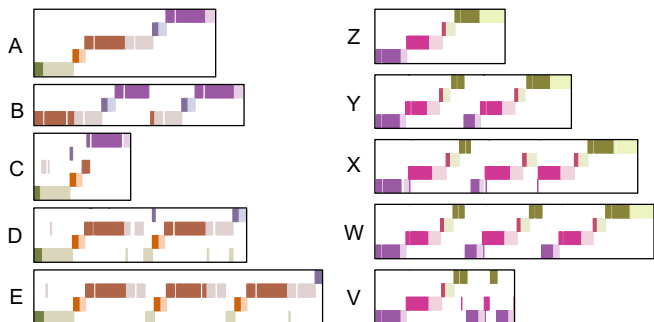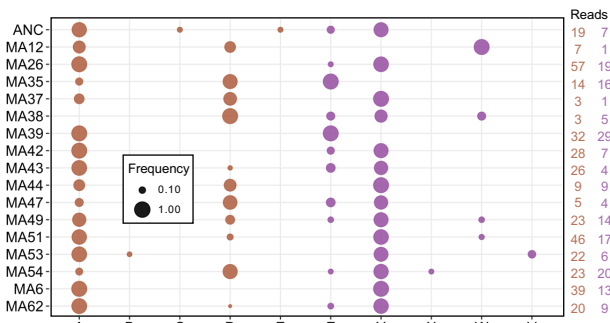

### 3D7 Reference

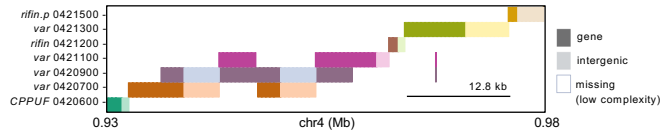

### 'Clonal' MA lines

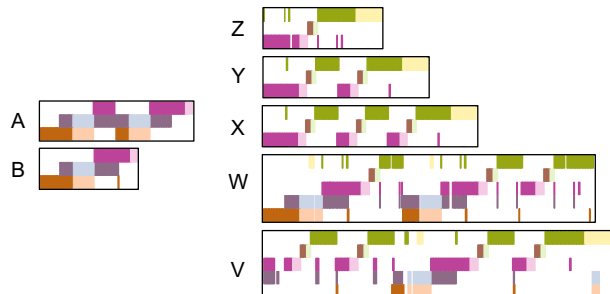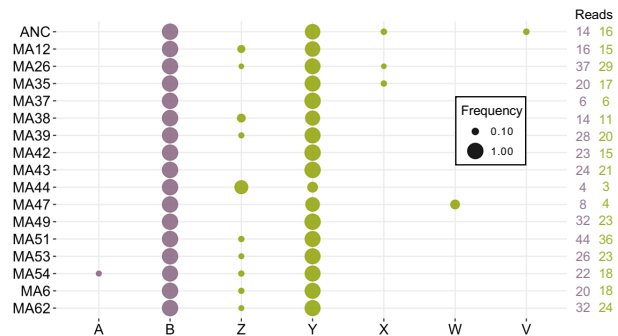

### 3D7 Reference

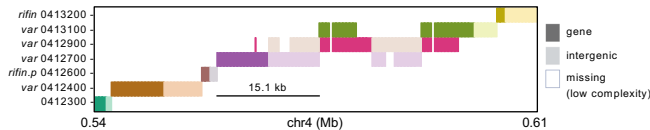

### 'Clonal' MA lines

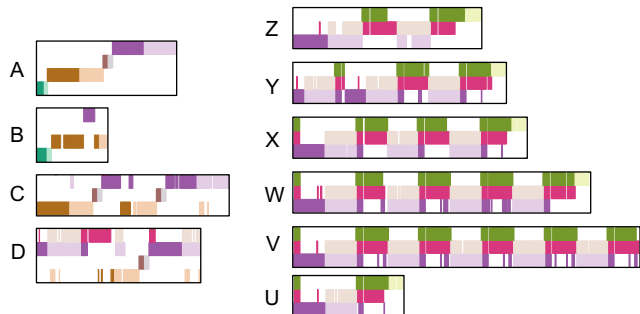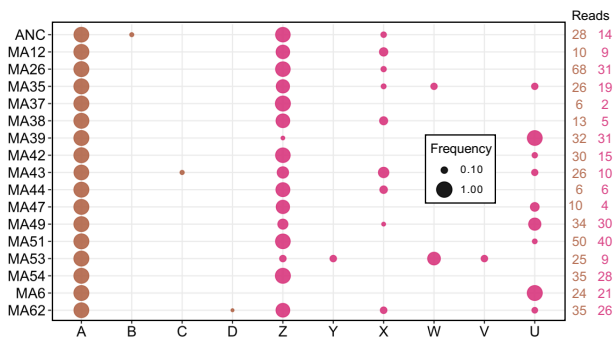

Supplement: Supplement 1 — Extended Data Fig. 1: Structural variation among assembled PacBio contigs. a, Genome-wide dot plots. Insets are internal var with >1 contig. b, Summary of loci with >1 contig across assemblies. c, Visualization of indel polymorphism across contigs at two internal var loci on chr4. Extended Data Fig. 2: Molecular confirmation of long-read polymorphism. a, PCR of breakpoints observed in contigs that map to the second internal var locus on chr12. Colored bands represent amplicons. Asterisks indicate the amplicons expected in each sample, based on assembled PacBio contigs. In the left diagram, the pink amplicon is expected to be 562 bp in the reference allele (e.g. allele A) and 667 bp with the gene conversion from PF3D7_0700100 (e.g. allele B). These data confirm the existence of breakpoints detected with PacBio in ANC and MA53 but undetected in MA39 and MA47. b, PCR detection of allele F. Asterisks indicate the amplicons expected in each sample, based on Nanopore reads. c, Southern blot of copy number variation in PF3D7_1240300. Teal asterisks mark the bands expected in each sample, based on Nanopore reads. Extended Data Fig. 3: Structural polymorphism across Nanopore reads mapping to internal var loci from all clonal MAL. Extended Data Fig. 4: Genome-wide coverage of extrachromosomal, circular DNA relative to genomic DNA. Extended Data Fig. 5: Large inverted duplications on ecDNA reads. a, Example read from MA54 containing a large inverted duplication. b, Plasmid-Safe-treated DNA is strongly enriched for “triangle reads”. c, Signal degradation consistent with single-strand annealing after passing through Nanopore. Supplementary Table 1. Summary of PacBio assemblies including PAF alignments to 3D7. Supplementary Table 2. ddPCR count data from QuantaSoft. Supplementary Table 3. Structural variation on Nanopore reads assigned to non-hypervariable var loci. The three events fixed in ANC and all MAL are considered wild-type. [file media-1.zip › supplement_biorxiv_020223/EDF3-Nanopore-chr7-chr4.pdf]

telomeric var internal var

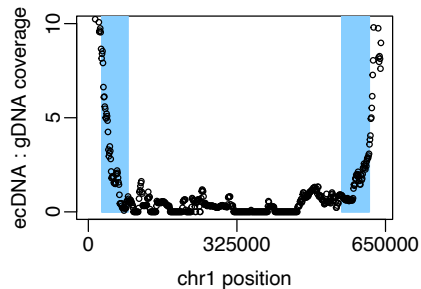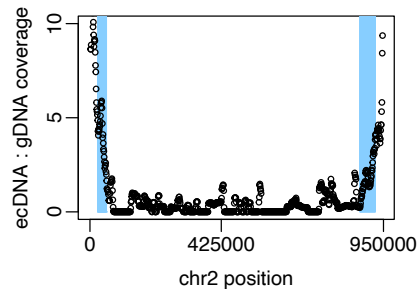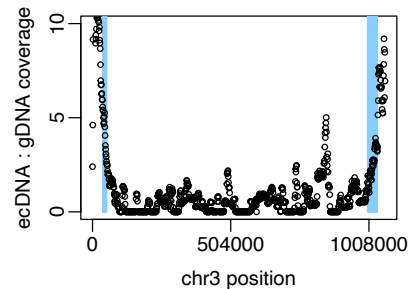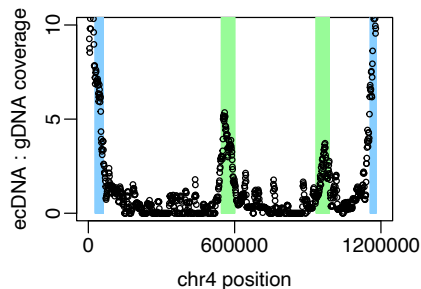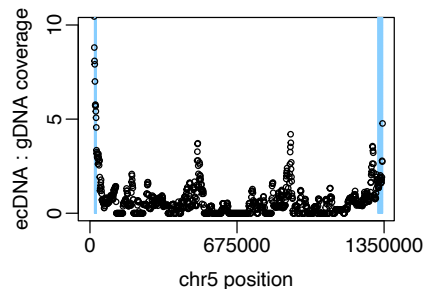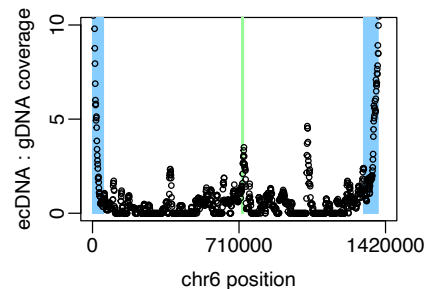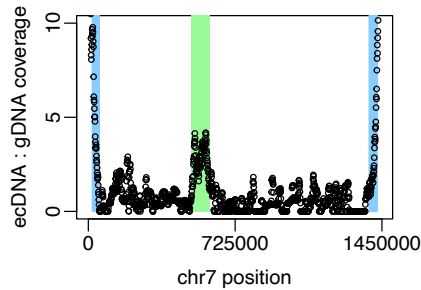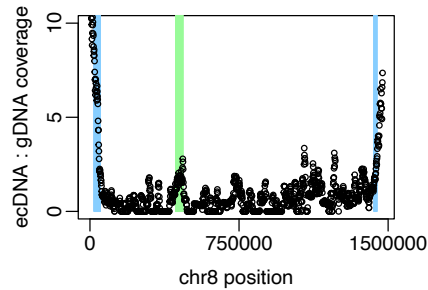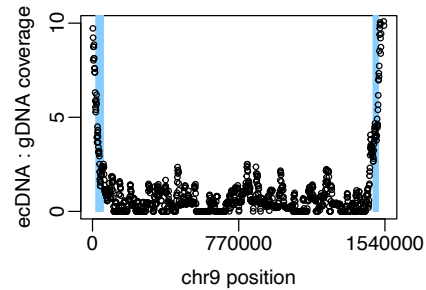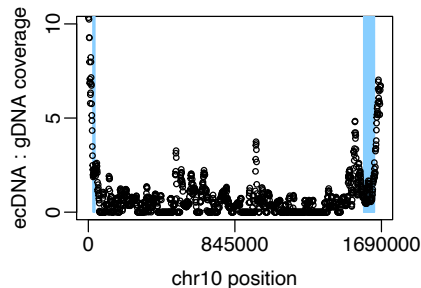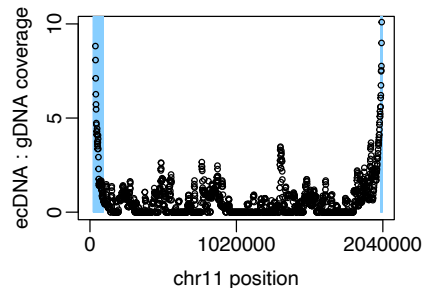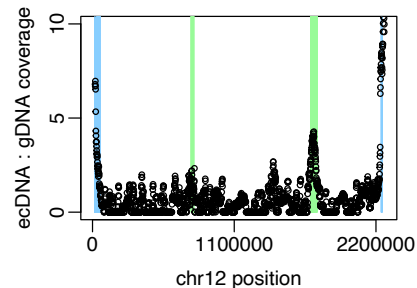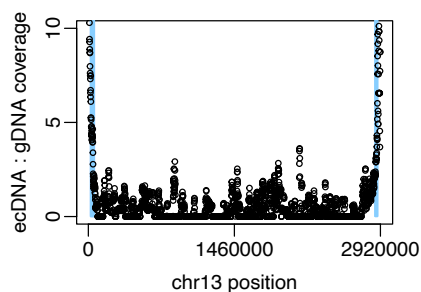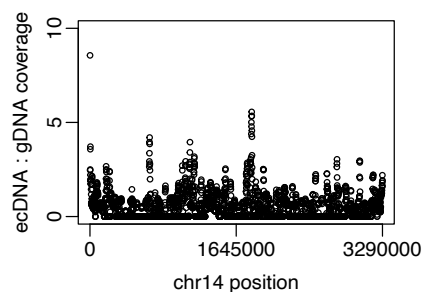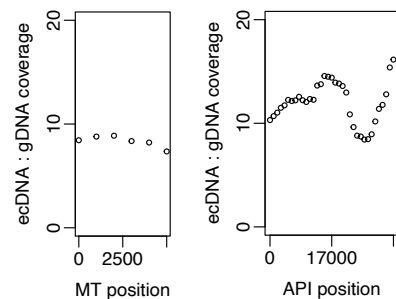

Supplement: Supplement 1 — Extended Data Fig. 1: Structural variation among assembled PacBio contigs. a, Genome-wide dot plots. Insets are internal var with >1 contig. b, Summary of loci with >1 contig across assemblies. c, Visualization of indel polymorphism across contigs at two internal var loci on chr4. Extended Data Fig. 2: Molecular confirmation of long-read polymorphism. a, PCR of breakpoints observed in contigs that map to the second internal var locus on chr12. Colored bands represent amplicons. Asterisks indicate the amplicons expected in each sample, based on assembled PacBio contigs. In the left diagram, the pink amplicon is expected to be 562 bp in the reference allele (e.g. allele A) and 667 bp with the gene conversion from PF3D7_0700100 (e.g. allele B). These data confirm the existence of breakpoints detected with PacBio in ANC and MA53 but undetected in MA39 and MA47. b, PCR detection of allele F. Asterisks indicate the amplicons expected in each sample, based on Nanopore reads. c, Southern blot of copy number variation in PF3D7_1240300. Teal asterisks mark the bands expected in each sample, based on Nanopore reads. Extended Data Fig. 3: Structural polymorphism across Nanopore reads mapping to internal var loci from all clonal MAL. Extended Data Fig. 4: Genome-wide coverage of extrachromosomal, circular DNA relative to genomic DNA. Extended Data Fig. 5: Large inverted duplications on ecDNA reads. a, Example read from MA54 containing a large inverted duplication. b, Plasmid-Safe-treated DNA is strongly enriched for “triangle reads”. c, Signal degradation consistent with single-strand annealing after passing through Nanopore. Supplementary Table 1. Summary of PacBio assemblies including PAF alignments to 3D7. Supplementary Table 2. ddPCR count data from QuantaSoft. Supplementary Table 3. Structural variation on Nanopore reads assigned to non-hypervariable var loci. The three events fixed in ANC and all MAL are considered wild-type. [file media-1.zip › supplement_biorxiv_020223/EDF4-ecDNA-coverage.pdf]
