# Supplementary material for: Antigenic diversity in malaria parasites is maintained on extrachromosomal DNA
Source: bioRxiv. 2023 Feb 2:2023.02.02.526885. Preprint. [Version 1] doi: 10.1101/2023.02.02.526885 (PMC9915586; doi:10.1101/2023.02.02.526885)
Supplement: Supplement 2 [file NIHPP2023.02.02.526885v1-supplement-2.pdf]

Extended Data Table 1. Probe and primer sequences. Colors refer to diagrams in EDF 2.

Experiment	Item	Forward primer/probe	Reverse Primer
PCR	Pink amplicon	TCAACCCAGACGACAACATC	AAAGTGCCTCGGTTGAGAC
PCR	Black amplicon	CAGATCCATGCAGACTTGTAGAGGATTA	GTATAGGCGCAACAGTTCCCAC
PCR	Red amplicon	CATCCGTGCGGAATAGGAAA	CTCACACAGGCATGTAACCA
PCR	Tan amplicon	GAAGAACTCTCCACAGAC	AGAGTGGTGACAAAGATATGT
PCR	Blue amplicon	ACCAAGTCATACCACAAGTGAA	GGTAACAAAGAACCTAGTGACGA
PCR	Gray amplicon	AAACTACGGTTGGAGGTGTG	AAGAGGAAACACAAGGACAGG
PCR	Yellow amplicon	AGATGACGACAACGAAGAAGAG	TGGCTTCAGCACCACTTT
Southern Blot	*var* probe	TGCCACGTTGTGAGTGGTAA	ATCAAGGCCCCCTTCAGGTA
ddPCR	*var* amplicon	CGCTTGGAAGTCAGGAAA	GTGGTGGTACAGTCGTTG
ddPCR	control amplicon	CGGCTCTTCGCATAGATT	GTGCCCTTGTATGGATCTG
ddPCR	*var* probe	AAATTGGTGAGTGCAACCGCTTCC	-
ddPCR	control probe	TGCTATCAATACACACGCATCAATAAACT	-
